# On the 3D Nature of the Magpie (Aves: *Pica pica*) Functional Hindlimb Anatomy During the Take-Off Jump

**DOI:** 10.3389/fbioe.2021.676894

**Published:** 2021-06-29

**Authors:** E. A. Meilak, N. J. Gostling, C. Palmer, M. O. Heller

**Affiliations:** ^1^Bioengineering Research Group, Faculty of Engineering and Physical Sciences, University of Southampton, Southampton, United Kingdom; ^2^Faculty of Environmental and Life Sciences, University of Southampton, Southampton, United Kingdom; ^3^Centre for Sport, Exercise and Osteoarthritis Research Versus Arthritis, Southampton, United Kingdom; ^4^Institute for Life Sciences, University of Southampton, Southampton, United Kingdom

**Keywords:** avian, hip, muscles, moments, biomechanics, magpie

## Abstract

Take-off is a critical phase of flight, and many birds jump to take to the air. Although the actuation of the hindlimb in terrestrial birds is not limited to the sagittal plane, and considerable non-sagittal plane motion has been observed during take-off jumps, how the spatial arrangement of hindlimb muscles in flying birds facilitates such jumps has received little attention. This study aims to ascertain the 3D hip muscle function in the magpie (*Pica pica*), a bird known to jump to take-off. A musculoskeletal model of the magpie hindlimb was developed using μCT scans (isotropic resolution of 18.2 μm) to derive bone surfaces, while the 3D muscle path definition was further informed by the literature. Function was robustly characterized by determining the 3D moment-generating capacity of 14 hip muscles over the functional joint range of motion during a take-off leap considering variations across the attachment areas and uncertainty in dynamic muscle geometry. Ratios of peak flexion-extension (FE) to internal-external rotation (IER) and abduction-adduction (ABD) moment-generating capacity were indicators of muscle function. Analyses of 972 variations of the 3D muscle paths showed that 11 of 14 muscles can act as either flexor or extensor, while all 14 muscles demonstrated the capacity to act as internal or external rotators of the hip with the mean ratios of peak FE to IER and ABD moment-generating capacity were 0.89 and 0.31, respectively. Moment-generating capacity in IER approaching levels in the FE moment-generating capacity determined here underline that the avian hip muscle function is not limited to the sagittal plane. Together with previous findings on the 3D nature of hindlimb kinematics, our results suggest that musculoskeletal models to develop a more detailed understanding of how birds orchestrate the use of muscles during a take-off jump cannot be restricted to the sagittal plane.

## Introduction

Take-off is a critical phase of flight, and many land birds perform some form of jump to take to the air. Although recent work strongly indicates that the hindlimbs are a key contributor to providing the initial take-off velocity (Heppner and Anderson, 1985; Bonser and Rayner, 1996; Earls, 2000; Tobalske and Dial, 2000; Tobalske, 2004; Henry et al., 2005; Berg and Biewener, 2010; Provini et al., 2012; Chin and Lentink, 2017; Provini and Abourachid, 2018), how exactly birds use their hindlimbs to take to the air has received little attention. Through their contraction, muscles act as “motors,” driving hindlimb motion, and therefore, understanding avian muscle function during the take-off jump is a first step to understand how the hindlimb contributes to taking to the air.

Current understanding of the functional anatomy of the avian hindlimb is informed by pioneering work that provides a detailed, but primarily qualitative, characterisations of muscle function based on anatomical dissection (Hudson, 1937; Wilcox, 1952; Verstappen et al., 1998; Smith et al., 2006). Whilst methods to quantitatively describe muscles’ function based on analyses of their 3D moment arms and moment-generating capacity are well established (Jensen and Davy, 1975; Murray et al., 1995; Hutchinson et al., 2005; Arnold et al., 2009; O’Neill et al., 2013; Charles et al., 2016), few studies applied these techniques to the avian hindlimb. Hutchinson et al. (2015) determined ostrich (*Struthio camelus*) muscle function based on the moment arms throughout the range of motion (RoM), defined by osteological joint congruency, a measure of how bones articulating at a joint relate to each other. Using that quantitative approach, the major function of the M. Obteratorius medialis (MOM) was identified to be that of a flexor muscle. In contrast to this view, Smith et al. (2006), whose definition of muscle function was determined by anatomical dissection, suggested that the main function of the MOM was that of an extensor. These opposing functional definitions for the same muscle in the same species demonstrate how the methodology for determining muscle function significantly affects the outcome. Additionally, investigation into the muscle function of theropods, the wider clade that avians belong to, yielded that all muscles in the hindlimb essentially act in all three rotational degrees of freedom, therefore, highlighting their inherent multi-functionality (Hutchinson et al., 2005; Hutchinson and Allen, 2009). Taken together, these findings suggest that the robust identification of muscle function in the avian hindlimb requires the use of 3D comprehensive sensitivity analyses (Modenese and Kohout, 2020).

Although a first description of the essential muscle function for the magpie (*Pica pica*), a bird known to jump to take to the air, is available in the literature (Verstappen et al., 1998), muscle function was estimated for only a limited number of muscles based on a 2D moment arm analysis in the sagittal plane. Initial analyses to characterise avian hindlimb motion used surface markers and investigated the sagittal plane motion only (Earls, 2000; Kambic et al., 2017). The introduction of novel technology has enabled the capture of 3D motion (Rubenson et al., 2007, 2010; Kambic et al., 2014, 2017; Provini and Abourachid, 2018). Such analyses have shown substantial motion not only in the sagittal plane, but also in the transverse and frontal planes during the avian take-off leap. It is therefore reasonable to assume that such motion must be either actively generated or at least controlled by the muscles. The 3D nature of avian take-off kinematics, along with the finding that theropod musculature is 3D and multifunctional described in the literature (Hutchinson and Allen, 2009; Provini and Abourachid, 2018; Allen et al., 2021), supports the hypothesis that avian hindlimb musculature is multifunctional throughout the take-off jump. However, no quantitative analysis of the 3D moment-generating capacity of the avian hindlimb for this important motion is available in the literature to substantiate this hypothesis.

This study aims to robustly ascertain muscle function based on the 3D moment-generating capacity of the pelvic muscles in magpie. Based on the evidence that the kinematics of avian locomotion in general and specifically during the take-off jump is not restricted to the sagittal plane, the hypothesis here is that pelvic muscles will not only be able to flex/extend the hip but be substantially three-dimensional. Specifically, the extent of the capacity to produce moments about the abduction/adduction (ABD) and int/external rotation axes was expected to be similar to the one about the flex/extension axis.

## Materials and Methods

### Model Development

A musculoskeletal model of the magpie hindlimb was developed based on the dedicated CT scans performed for this study, histological samples of muscle cross-sections (Hudson, 1937), and bone attachment site sketches available from the literature (Verstappen et al., 1998). The CT data provided the basis for establishing a 3D surface model of the skeletal anatomy of the hindlimb. The definition of joint centres and axes and local bone coordinate systems are available in the Supplementary Information. Muscles were modelled by 3D lines of action (Heller et al., 2001b; Seth et al., 2011; Trepczynski et al., 2012) by relating the cross-sectional and attachment data to the bone surfaces. Muscle function was then robustly characterised by determining the 3D muscle moment arms and moment-generating capacity over the functional joint RoM during a take-off leap, whilst considering the key sources of uncertainty in the definition of static and dynamic muscle geometry. The study was approved by the University of Southampton Ethics Committee (ERGO ID 21781).

### Skeletal Model

A magpie *Pica pica* cadaver (190 g), mounted in a perched position in a clear acrylic cylinder and stabilised by floral foam (OASIS, Kent, OH, United States), was CT scanned at an isotropic resolution of 18.2 μm [225 kVp/450 kVp Nikon/Metris (Tokyo, Japan)], in a custom designed micro-focus computed tomography scanner (housed within the μ-VIS X-ray Imaging Centre, University of Southampton, United Kingdom) to capture the bone geometry. A threshold based semi-automatic segmentation followed by a marching cubes algorithm surface reconstruction (Hege et al., 1997) was used to obtain bone surfaces for the right hindlimb (Avizo 9.0.1, Thermo Fisher Scientific, Waltham, MA, United States). All surfaces were re-meshed using the isotropic re-meshing tool [Open Flipper 3.1 (Möbius and Kobbelt, 2012)] to a target mean triangle edge length of 0.1 mm. The skeletal structures identified in that manner included the pelvis, femur, patella, tibiotarsus and fibula, tarsometatarsus, and all phalangeal bones.

**FIGURE 1:**
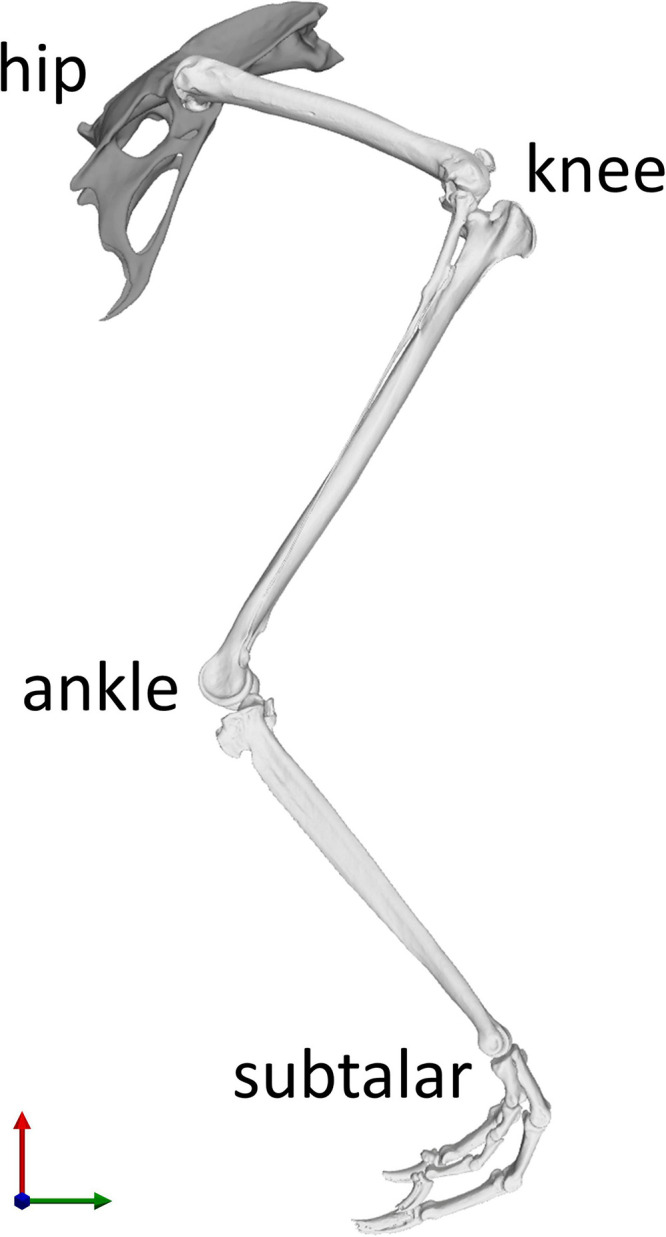
Skeletal model of the right hindlimb of the magpie in the reference position of the joints taken as the mean RoM of every joint degree of freedom (DoF). Each of the four joints (hip, knee, ankle, and subtalar) possesses three rotational DoFs. The knee joint incorporated patella-femoral kinematics which were expressed as a function of knee flexion (Trepczynski et al., 2012). The X axis direction (green) points from caudal to cranial, the Y axis direction (red) points from ventral to dorsal, and the Z axis direction (blue) points from left to right.

In order to establish a musculoskeletal model for further analysis, a linked rigid body model with six segments including the pelvis, thigh, shank, tarsometatarsus, and digits was defined in OpenSim v4.0 (Seth et al., 2018). Here, body segments were linked by four joints (hip, knee, ankle, and subtalar joints) with three rotational degrees of freedom each (Figure 1).

### Muscle Geometry

**FIGURE 2:**
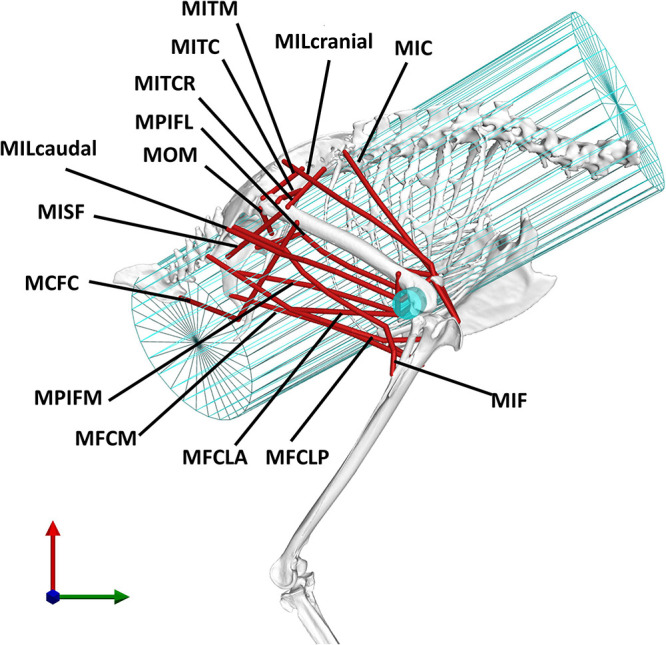
The analysis considered 14 key pelvic muscles of the magpie. To approximate the 3D paths of the muscle between origin and insertion, two wrapping cylinders (shown in blue) were introduced. The wrapping cylinder used for the MIC, simulating the action of the rib cage, is shown in a wireframe representation while the wrapping object used to simulate the interaction with the distal femoral condyles is shown by a solid cylinder. For an explanation of the abbreviations of the muscles used here, please refer to the key provided in Table 1.

**FIGURE 3:**
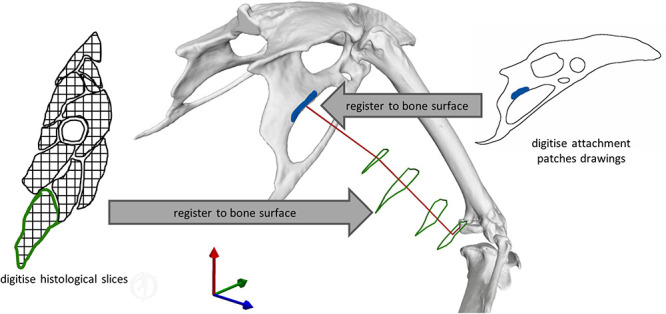
Illustration of the key principles for defining muscle geometry using literature data to support the definition of both muscle attachments and 3D muscle path geometry. *Right* muscle attachment patches from the literature (Verstappen et al., 1998) were digitised, and then registered to the respective bone surface. *Left* histological slices of the crow (*Corvus corone*) hindlimb from the literature (Hudson, 1937) were digitised, and then registered to the magpie bone surfaces. For each of these cross-sections, the centroids of the muscle contours were determined to inform the definition of the 3D muscle path spanning origin and insertion.

**FIGURE 4:**
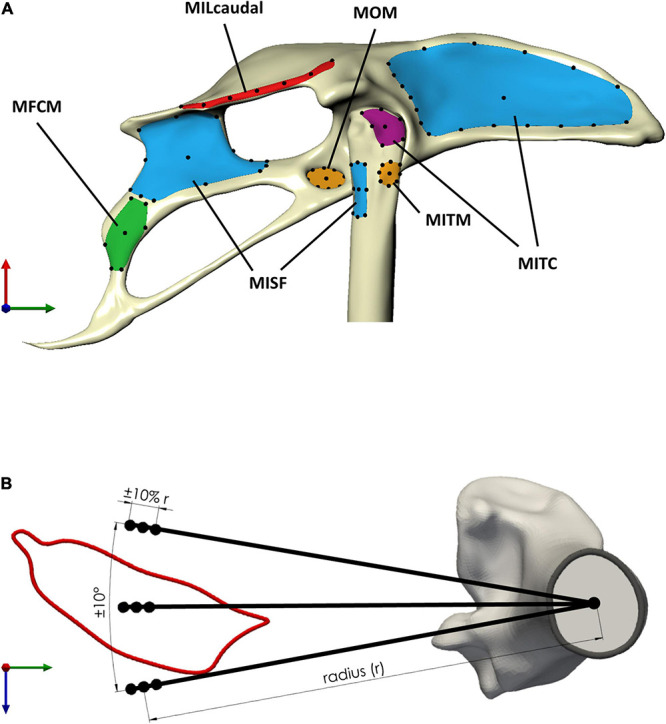
Sensitivity of the moment-generating capacity of the pelvic muscles was investigated with respect to uncertainty in the definition of muscle attachments and via points. **(A)** Muscle attachment shape was classified as either thin rectangular (red attachment area, MILcaudal_pelvis_), rectangular (green, MFCM_pelvis_), triangular (violet, MITC_femur_), or circular (orange, MOM_pelvis_ and MITM_femur_) (Table 2). Long thin attachments were discretised using seven points (MILcaudal_*femur*,_ black dots) along the medial axis of the attachment surface, while for attachments with other shapes, the discretisation considered the perimeter and centroid of the attachment area. For muscles with a larger perimeter (blue attachment area, MITC_pelvis_, MISF_pelvis_, and MISF_femur_), a larger number of points was defined on the perimeter. **(B)** Uncertainty in the definition of via points, as exemplified here for a via point for MFCLP_femur_, considered circumferential variation around the long bone axis by ±10° and radial variation by ±10% of the distance of the muscle contour centroid from the associated centroid of the bone cross-section (radius r).

Fourteen key hip muscles were modelled as polylines spanning origin and insertion while via points were added to fully describe their 3D paths (Figure 2). Outlines of the muscle attachment areas on the pelvis and femur, as known from the literature, (Verstappen et al., 1998) were re-traced on the surface models of the bones of the magpie specimen scanned here (Figure 3). To that end, between 30 and 35 landmarks were defined for each attachment area [MorphoDig v 1.5.3, Lebrun (2018)]. At the proximal femur in particular, prominent ridges on the bone surface further guided the delineation of the muscle attachments. The locations of the landmarks delineating the muscle attachment boundaries were imported into Rhino [v7; Robert McNeel & Associates, Seattle, United States (McNeel, 2020)] where closed polylines were created and rebuilt to obtain smooth curves. The triangulated bone surfaces were fitted by subdivision surfaces using the QuadRemesh function in Rhino before converting them to Non-uniform rational basis spline (NURBS) surfaces. The smooth curves outlining the muscle attachments on the femur and pelvis were then projected on the respective NURBS surface, providing a detailed description of muscle attachment geometry (Figure 4A). Further attachment patches on the shaft of the long bones were digitised from the literature (Verstappen et al., 1998) and projected onto the 3D bone surfaces of the specimen using polar coordinate system mapping techniques. The shaft of the long bones was approximated by a cylinder onto which the medial and lateral sketches of the attachment sites were projected.

The use of via points is considered important to better replicate a muscle’s curved path as differences in a muscle’s moment arms by up to 50% compared to being modelled as a straight line have been reported (Jensen and Davy, 1975; Modenese and Kohout, 2020). Histological slice images of the crow (Corvus corone, being a closely related member of the same family as the magpie) hindlimb, from which, such via points could be derived, were thus digitised and mapped onto the magpie hindlimb to establish the 3D muscle path. Registration of the histological slices to the bone was carried out by determining their relative location along the long bone axis and registering the cross-sections by fitting circles to both the crow and magpie data (Figure 3). For each slice, the histological soft tissue contours were scaled by the ratio of the radius of the crow to the magpie femoral radius in the respective cross-sections. The mapped data was further orientated with respect to the femur by Iterative Closest Point (ICP) registration of the distal slices containing the distal femoral condyles (Figure 3). Centroid locations of registered muscle contours were used to define the 3D muscle paths (Jensen and Davy, 1975; Duda et al., 1997, 2006; Allen et al., 2017). Wrapping cylinders and ellipsoids were added to define the 3D paths throughout the RoM to ensure that muscles do not intersect bones. Axes’ directions and radii of these wrapping objects were determined by least-squares fitting to selected regions of the bone surfaces (Figure 2).

### Muscle Moment-Generating Capability Analysis

In order to ascertain the muscle function, the study focused on the 3D moment-generating capacity of 14 pelvic muscles at the hip during the take-off jump. Moment arms of each muscle were assessed over the RoM of each rotational degree of freedom (DoF) at the hip [hip flexion/extension (FE), ABD, and internal/external rotation (IER)]. The RoM studied was based on the 3D skeletal kinematics of the take-off jump obtained from XROMM of the Diamond Dove and Zebra Finch (Provini and Abourachid, 2018). Here, the joint neutral pose was matched to the joint orientation published by Provini and Abourachid (2018). The RoM was calculated by taking the upper and lower limits of the mean joint angles of the take-off jumps of both species. The RoM about the FE, IER, and ABD axes derived in that manner were 54° (–62° to –8°), 20° (18°–38°), and 8° (–33° to –25°), respectively. Moment arms were determined in 1° increments analysing a single DoF at a time while the two DoFs which were not being assessed were set at their mean value of the RoM.

In order to derive the moment-generating capacity for each muscle, the maximum isometric force (*F*_max_*_,i_*) of the muscles was estimated first by relating the physical cross-sectional area (PCSA) to the maximum isometric stress under maximal activation (Eq. 1). Here, σ_*max*_ was taken as 3.0 × 10^5^ Nm^–2^ (Hutchinson, 2004; Nelson et al., 2004; Hutchinson et al., 2015; Rankin et al., 2016).

(1) $$F_{max,i}=PCSA_{i}\times \sigma_{\text{max}}$$

The moment-generating capacity of each muscle was then estimated using the PCSA data of a magpie [Verstappen et al. (1998), Eq. 2] matched to the current specimen by scaling by mass. For the purpose of the study, these moments were evaluated for each muscle *i* (where *i* = 1.14) for the muscle maximum isometric force (*F*_max_) at the mean moment arm ($\bar{\text{MA}}$) determined over the RoM for each rotational DoF *j* (where *j* = 1.3) of the hip joint.

(2) $$M_{i,j}=F_{\max,i}\cdot {\bar{\text{MA}}}_{i,j}$$

Attribution of muscle function was based on the moment-generating capacity of a muscle expressed as a percentage of the sum of the moments of all muscles acting in the same direction of the respective DoF:

(3) $$\frac{M_{i,j}}{\sum_{i=1}^{14}M_{i,j}}$$

Muscles were considered to contribute to a certain function (flexion, extension, abduction, adduction, and internal or external rotation) if a lower bound on their muscle moment-generating capacity, defined as the mean moment across all conditions minus 1 standard deviation, was greater than 2% of the sum of the mean moments of all muscles acting in the same direction.

### Sensitivity Analysis

In order to ascertain the robust estimates of muscle function when representing muscles by single lines of actions, two key sources of variability were considered in a sensitivity analysis, the location of muscle attachments and the position of the via points. The sensitivity analyses firstly considered a selection of the possible attachment locations for each attachment site. The selection was informed by the general shape and size of the muscle attachments. Circular attachments (Table 2) were represented by a single location at the position of the projection of the geometric centroid of the attachment onto the respective surface. Eight additional points were added on the perimeter placed at cardinal and intercardinal positions (Table 2). For larger muscle attachments with a more triangular or rectangular shape, the respective edges as well as the projection of the geometric centroid of the attachment patch onto its surface were all considered in the analyses (Table 2). For the largest attachment (MITC pelvis attachment), two additional, equidistantly distributed positions between the edges were considered while for the pelvic attachment of the MISF, the midpoints between edges were additionally considered. Most attachments on the pelvis were of a rectangular shape with a rather small height (ventro-dorsal) compared to their width (cranio-caudal) (Table 2). For these attachment surfaces, a medial axis was first determined [Rhino v7; Robert McNeel & Associates, Seattle, United States, (McNeel, 2020)], along which, then, a total of seven equally distant points were defined that were considered for the analyses (Figure 4A).

**TABLE 1:** This study considered 14 key pelvis muscles for further analysis, listed here in alphabetical order.

**Abbreviation**	**Muscle name**
MFCLA	M. flexor cruris lateralis pars accessoria
MFCLP	M. caudofemoralis pars caudalis
MFCM	M. flexor cruris medialis
MIC	M. iliotibialis cranialis
MIF	M. iliofibularis
MILcaudal	M. iliotibialis lateralis caudalis
MILcranial	M. iliotibialis lateralis cranialis
MISF	M. ischiofemoralis
MITC	M. iliotrochantericus caudalis
MITCR	M. iliotrochantericus cranialis
MITM	M. iliotrochantericus medius
MOM	M. obteratorius medialis
MPIFL	M. puboischiofemoralis pars lateralis
MPIFM	M. puboischiofemoralis pars medialis

**TABLE 2:** Muscle attachment sites were grouped by their shape which determined how they were discretized (number of points) for the sensitivity analysis.

**Shape**	**Muscle**	**Number of points**
**Thin rectangular**		
	MFCLA_pelvis_	7
	MFCLP_pelvis_	7
	MIC_pelvis_	7
	MIF_pelvis_	7
	MILcaudal_pelvis_	7
	MILcranial_pelvis_	7
	MITCR_pelvis_	7
	MITM_pelvis_	7
	MPIFL_pelvis_	7
	MPIFM_pelvis_	7
**Rectangular**		
	MFCM_pelvis_	7
	*MISF_pelvis_	15
	*MISF_femur_	9
**Triangular**		
	*MITC_pelvis_	19
	MITC_femur_	7
	MITCR_femur_	7
**Circular**		
	MITM_femur_	9
	MOM_pelvis_	9
	MOM_femur_	9

*Subscripts denote which bone a muscle is attached to. For muscles with a larger perimeter (indicated by asterisk), a larger number of points was considered.*

To assess how the uncertainty in the definition of path points affected the moment-generating capacity, via points could vary radially and circumferentially from their initial position at the centroid of the muscle cross-sections. Estimates of the variation in muscle paths across a wide range of bird species were obtained to derive informed limits on how much the via point locations could vary circumferentially. For 11 muscles of the hip, histological slices covering the cross-sectional musculoskeletal anatomy of a variety of bird species (sparrow hawk *Falco sparverius*, Screech owl *Otus asio*, Green heron *Butorides virescens*, and Lesser yellowlegs *Totanus flavipes*) (Hudson, 1937) were digitised and related to the bones as described above. The centroids of the muscle cross-sections were transformed to polar coordinate systems, with the origin located at the centroid of the femoral cross-section of the magpie specimen to then determine the standard deviation of their angular positions. The standard deviation of the angular position of these 11 muscles across five species was 10°. Therefore, via point locations were varied circumferentially by ±10°.

The intraspecies variation in the physiological cross-sectional area (PCSA) was used to derive an upper limit for how much the locations of the via points could vary radially. Based on the consideration that the radial distance of the centroid of a muscle with a larger PCSA would be further away from the bone, the extent of PCSA variation was taken as a proxy for the extent of variation in the radial position. A study into the variability in the muscle architecture of the Monk Parakeet (*Myiopsitta monachus*) showed that the maximum coefficient of variation in the PCSA of a muscle was approximately 10% (Carril et al., 2014). Therefore, a variation in the radial direction of the via point location of ±10% of the reference value was considered (Figure 4B).

For each muscle, the mean, standard deviation, and coefficient of variation of its moment-generating capacity were computed at each joint angle increment (1°) for all possible combinations of origin, via point, and insertion locations as appropriate. From these, the mean moment and coefficient of variation (CV) were calculated across the entire RoM and used as a measure of the muscle moment-generating capacity and how robustly the function of a muscle was ascertained, respectively. As an overall measure of change in the muscle moment-generating capacity throughout the functional RoM, the percentage change of each muscle’s moment arm over the RoM per DoF was further calculated.

In order to assess the influence of the way in which the 3D muscles paths were modelled, muscles were classified to belong to either of two categories based on the mean CV of their moment arms throughout the functional RoM. With a view to differentiate modelling artefacts from genuine changes in muscle moment arms throughout the RoM, muscles with a substantial CV (operationally defined here to be greater than 50%) were considered further (Heller et al., 2001b, 2005; Trepczynski et al., 2012, 2018; Charles et al., 2016). A single muscle’s moment arm can differ by up to 50% depending on whether it is modelled as a straight line, spanning origin and insertion site, or follows the 3D curved centroid line of the muscle (Jensen and Davy, 1975; Modenese and Kohout, 2020). Therefore, a CV of over 50% indicates that a muscle’s variation in moment arms is not only due to the modelling approach but due to actual changes in muscle moment arms over the RoM. To quantify the contribution of modelling individual path points to the sensitivity of the muscle moment arms, locations of origin sites and via points were varied independently and CVs were calculated for each condition and compared. The peak value of the ratios comparing moment arms in IER and ABD to FE were determined as a measure to quantify a muscle’s potential to actuate degrees of freedom outside of the sagittal plane. Moments of all muscles acting in the same direction were summed before calculating the respective ratios.

## Results

**FIGURE 5:**
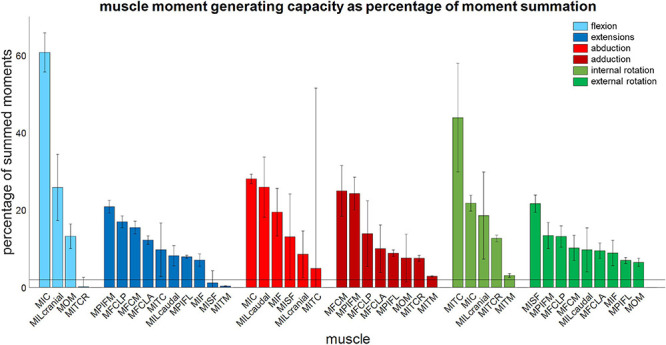
Muscle moment-generating capacity as a percentage of the sum of all moments acting in the same direction. Error bars show ±1 standard deviation. Muscles for which the mean moment-generating capacity minus 1 standard deviation was less than 2% of the sum of all moments were not considered to have a function about that axis.

**FIGURE 6:**
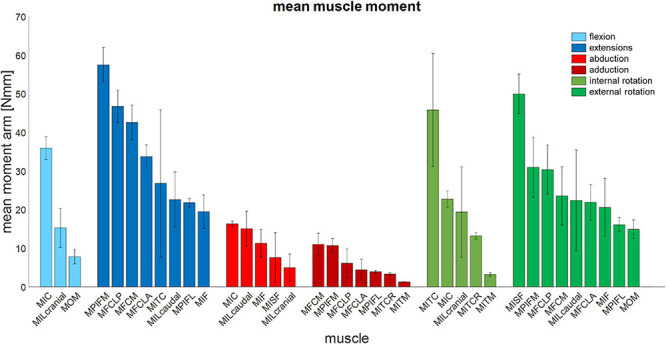
Mean muscle moment-generating capacity of 14 key pelvic muscles of the magpie about all three rotational degrees of freedom of the hip. Error bars show ±1 standard deviation.

The moment-generating capacity of 14 muscles was successfully analysed over the functional hip joint RoM of a take-off leap for a total of 972 variations of 3D muscle paths. Nine muscles were found to have functions about all three rotational DoFs, and the remaining five about only two DoF (Table 3 and Figure 5). No muscle had a function restricted to just the transverse plane (flex/extension action). Moreover, three muscles (MITM, MITCR, and MISF) produced significant moments only about the ABD axis and int/external rotation axis and not about the flex/extension axis (Table 3 and Figure 5). Every muscle had the ability to generate either internal or external rotations to the femur. All of the 11 muscles with capability to act as flexor or extensor had a similar capability to act as internal/external rotators, evidenced by a mean ratio of peak IER to FE moments of 0.89 ± 0.33. Conversely, the ability of flexors and extensors to act as abductors and adductors was considerably less, with a mean ratio of peak ABD to FE moments of 0.31 ± 0.19. Moments that acted to extend and externally rotate the hip made up most of the sum of the moment-generating capacity of all pelvic muscles (36 and 30%, respectively). The largest moment-generating capacity was observed for the extensors and external rotators, with values of 57.5 ± 4.5 and 50 ± 5.1 Nmm for the MPIFM and MISF, respectively (Figure 6).

**TABLE 3:** Function of key pelvic muscles of the hindlimb of the magpie as ascertained in this study for flexion/extension (F/E), abduction/adduction (AB/AD), and internal/external rotation (Int/Ext) were compared to data from the literature (Verstappen et al., 1998).

**Muscle**	**F/E**		**AB/AD**	**Int/Ext**
	**This study**	**Literature**	**This study**	**This study**
MFCLA	E	E	AD	E
MFCLP	E	E	AD	E
MFCM	E	E	AD	E
**MIC**	**F**	**O**	**AB**	**I**
MIF	E	E	AB	E
**MILcaudal**	**E**	**O**	**AB**	**E**
**MILcranial**	**F**	**O**	**AB**	**I**
MISF		O	AB	E
**MITC**	**E**	**F**		**I**
**MITCR**		**F**	**AD**	**I**
**MITM**		**F**	**AD**	**I**
MOM	F	F		E
MPIFL	E	E	AD	E
MPIFM	E	E	AD	E

*The letter O indicates conditions where Verstappen et al. (1998) attributed a muscle a function about a joint other than the hip. Muscles found to have a function that differs from that reported by Verstappen et al. (1998) are highlighted in bold.*

Throughout the RoM, the mean change in moment arms for most muscles was 24 ± 20% excluding the MOM and MIC for which maximum moment arm changes around the FE axis and ABD of up to 106 and 150%, respectively, were observed.

The largest CVs of all muscles were observed for the MITC, MISF, MILcaudal, MILcranial, and MFCLP/MFCLA with values ranging between 59 and 85% (Table 4). The CVs for the MISF and MITC were the largest, with mean CVs of 85 and 77% about the ABD and flex/extension axes, respectively. The MILcranial had a mean CV of 70% about the int/external rotation axis whereas the MFCLP and MFCLA, both originating from the same location on the pelvis and possessing an adjacent initial via point on the femur, exhibited a mean CV of 62% about the ABD axis. Similarly, the MILcaudal had a mean CV of 59% about the ABD axis. Upon further investigation of the contribution of modelling individual path points to the sensitivity of the muscle moment arms, it was revealed that the moment arms of the MITC, MISF, MILcaudal, and MILcranial muscles were most sensitive to variations in the location of the origin sites. Here, mean CVs ranged between 61 and 316% due to the variability of the origin site compared to 40–99% mean CVs from varying the via point (Table 5). The moment arms of the MFCLP and MFCLA were most sensitive to how the path point was varied, with mean CVs ranging from 16 to 41–43% due to the variability in origin sites and via points, respectively (Table 5). The CVs for the remaining eight muscles (MFCM, MIC, MIF, MITCR, MITM, MOM, MPIFL, and MPIFM) remained between 6 and 37% (Table 3).

**TABLE 4:** The mean coefficient of variation (CV) of each muscle’s moment-generating capacity computed at each increment of joint angle from all possible combinations of origin, via point, and insertion locations as appropriate.

**Muscle**	**F/E (%)**	**AB/AD (%)**	**Int/Ext (%)**
MFCLA	9	62	21
MFCLP	9	62	21
MFCM	11	26	33
MIC	9	4	9
MIF	23	32	37
MILcaudal	33	30	59
MILcranial	33	70	61
MISF		85	10
MITC	77		32
MITCR		10	7
MITM		6	15
MOM	25		16
MPIFL	5	9	11
MPIFM	8	17	25

*Blank cells indicate conditions where a muscle does not have a function with respect to the respective degree of freedom.*

**TABLE 5:** Mean CV of the muscle moment-generating capacity in response to varying origin site and via point locations.

	**Mean CV**	
**Muscle**	**Origin (%)**	**Via point (%)**
MFCLA_AB/AD_	16	**41**
MFCLP_AB/AD_	16	**43**
MILcaudal_Int/Ext_	**61**	40
MILcranial_AB/AD_	**72**	45
MISF_AB/AD_	**81**	63
MITC_F/E_	**316**	99

*Only muscles for which the mean CV was particularly large (over 50%) were investigated further (MFCLA, MFCLP, MILcaudal, MILcranial, MISF, and MITC). For these muscles, further analyses revealed whether the moment-generating capacity was most sensitive to varying either the origin site or via point locations. Subscripts denote the axis about which the moment-generating capacity was most sensitive. Values highlighted in bold indicate which of the muscle’s origin or via point had the greatest influence on the muscles’ moment generating capacity.*

## Discussion

**FIGURE 7:**
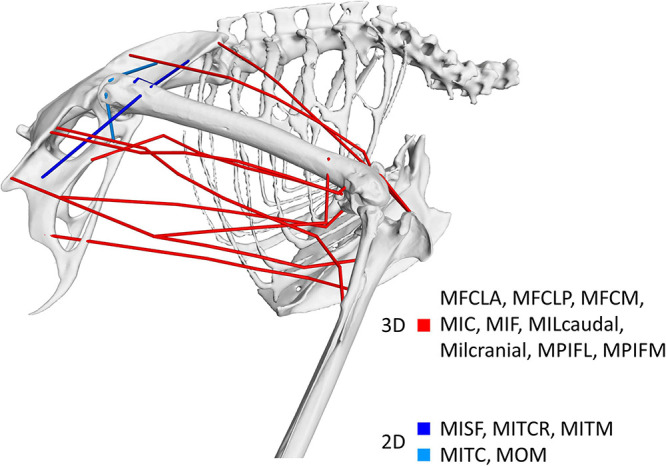
Visual representation of the nature of the 3D muscle function identified in this study. Muscles shown in red were found to possess dominant moment arms in 3D while those shown in blue had functions around two axes only. Muscles shown in dark blue represent those whose functions are about the ab/adduction and int/external rotation axes while the function of muscles depicted in light blue is about the flex/extension and int/external rotation axes.

This study aimed to quantify the 3D function of the pelvic muscles of the magpie (*Pica pica*) based on the muscle moment-generating capacity throughout the take-off jump. Based on a previous description of the 3D nature of the kinematics of bird jumping take-offs (Provini and Abourachid, 2018), the underlying hypothesis was that pelvic muscles were multi-functional rather than acting solely in the sagittal plane. Our analyses show that although 11 of the 14 key pelvic muscles investigated here do indeed act in the sagittal plane where they function as either flexors or extensors, all 14 muscles also act as either internal or external rotators (Figure 7). The 3D muscle moment-generating capacities determined here indeed reveal that internal/external moments are of similar magnitude to those in FE with the mean ratio between a muscle’s peak IER and FE of 0.89 ± 0.33. This capacity of the pelvic muscles to actuate 3D moments reported here is consistent with the kinematic analyses that characterise the motions as 3D rather than planar (Provini and Abourachid, 2018). Additionally, our findings agree with the notion that theropod hindlimb muscles are generally more multifunctional (Hutchinson and Allen, 2009), possessing moment-generating capacity about all 3 joint degrees of freedom. The 3D nature of the kinematics and the moment-generating capacity of the muscles suggest that in order to develop a detailed understanding of avian take-off mechanics, hindlimb anatomy should be considered in 3D.

In the past, muscle function in birds has been ascertained primarily through the anatomical dissection of muscle-tendon paths (Hudson, 1937; Wilcox, 1952; Verstappen et al., 1998; Smith et al., 2006) and remained limited to function about the flexion extension axis for single joints, including muscles which cross multiple joints. Muscle function about FE determined by the quantitative approach here agreed with the literature for all muscles except for the MITC which had previously been defined as a flexor but was defined here as an extensor (Table 3). The MITC is a muscle with a large attachment area at its pelvic origin, inserting proximal to the femoral neck. Depending on the level of ABD and IER, the muscle line of action could either be above or below the FE axis of rotation. Therefore, it is possible for the function of the muscle about the FE axis to swap depending on the orientation of the femur. Although the MITC’s moment-generating capacity was sensitive to the modelling process (Table 3), the function never swapped from extensor to flexor in our analyses when considering the joint RoM for a take-off leap previously determined by 3D fluoroscopy (Provini and Abourachid, 2018). The current study thus provides evidence that throughout the jump, the activity considered here, the MITC acts exclusively as a hip extensor.

The literature describes three muscles attaching at the pelvis (MIC, MILcaudal, and MILcranial) to have a function about the knee but does not report about their role at the hip (Table 3; Verstappen et al., 1998). Our analyses showed that the MIC and MILcranial contributed substantially to the moment-generating capacity in hip flexion, accounting for 61 and 26%, respectively, to the overall capacity (Figure 5 and Table 3) and thus point toward a crucial role of these muscles for hip function also. On the other hand, the MILcaudal accounted for only 8% of the total extensor moment-generating capacity, suggesting a more limited role for its hip function during a jump.

During the take-off jump, all joints of the hindlimb, including the knee, are extended through activation of the muscles. The hip flexors MIC and MILcranial also have the capacity to extend the knee (Verstappen et al., 1998) and if activated during take-off, would work against the hip extensors. Such seemingly paradoxical muscle function may add to the hip joint stability during the jump by active co-contraction, a mechanism that has been described for human hindlimb (Herzog and Binding, 1993; Ait-Haddou et al., 2000; Jinha et al., 2006a, b; Correa et al., 2010; Trepczynski et al., 2018). Further studies using computational modelling approaches to either estimate muscle activation patterns (Rankin et al., 2016) or work to directly measure muscle activation (Higham et al., 2008) are required to further elucidate the nature of avian hindlimb muscle coordination patterns during a take-off leap.

It is well established that the muscle moment-generating capacity determined by a model is sensitive to how the 3D muscle lines of actions are described (Jensen and Davy, 1975; Blemker and Delp, 2005; Heller et al., 2005; O’Neill et al., 2013; Charles et al., 2016). The current study took a rigorous approach to estimate the extent of uncertainty in defining the 3D muscle paths considering likely morphological variation in muscle attachments and via points within the family of corvids and, more widely, across extant birds (Hudson, 1937; Verstappen et al., 1998; Carril et al., 2014). Our sensitivity analysis considering 972 different configurations of the 3D muscle paths demonstrated modest variation in the moment-generating capacity for 8 of the 14 hip muscles (mean CV below 50%) suggesting that modelling their function by a single line of action is appropriate (Monti et al., 2001; O’Neill et al., 2013; Charles et al., 2016; Modenese and Kohout, 2020).

In contrast, the moment-generating capacity of the MITC, MISF, MILcranial, MILcaudal, MFCLP, and MFCLA varied between 59 and 85% (Table 4). The MITC, MISF, MILcranial, and MILcaudal are muscles that have large attachment areas on the pelvis with substantial cranio-caudal extent and, depending on the location of a modelled muscle line of action, their moment-generating capacity at the hip may vary considerably. Modelling muscles with large attachment sites with multiple lines of action is common practice when modelling the biomechanics of the human hip, where the glutei are typically modelled by three distinct lines of action (Arnold et al., 2009; Higham and Biewener, 2011; Modenese and Kohout, 2020). The analyses here suggest that a similar approach would also be beneficial to capture the varied function of the MITC, MISF, MILcranial, and MILcaudal in the avian hindlimb. On the other hand, the large moment arm sensitivity of the MFCLP/MFCLA was mainly due to the extent of the variation of the via points considered here, with circumferential variation of 10 degrees, a value determined from the variation of 11 hip muscles across the five species considered. However, for these specific muscles, the actual variation between species was considerably smaller with only two degrees suggesting that the sensitivity determined here represents a safe upper bound for the likely effect.

Muscle function in the avian hindlimb has previously been examined using similar, quantitative techniques throughout the full RoM of the joints of the ostrich, an extant flightless bird (Hutchinson et al., 2015). Whilst the exact details and extent to which pelvic muscles in the ostrich take on a 3D function appear to vary somewhat from the data reported here, Hutchinson et al. (2015) provided a strong evidence for the function of pelvic muscles in the ostrich to be 3D in nature rather than being limited to the sagittal plane, where all pelvic muscles were found to have functions about all three rotational degrees of freedom, and four muscles (IC, ILp, FCLP, and OM) were found to have a substantial capacity to act about multiple degrees of freedom at the hip. Even though the ostrich and magpie sit on opposite ends of the phylogenetic tree (Jarvis et al., 2014), the functional demand from their habitual locomotor activities (running and jumping) appears to necessitate hip muscle function in flightless and flying birds alike to be 3D in nature.

Extending the methodology established here to robustly quantify the 3D muscle function at the hip to muscles crossing the knee and ankle constitutes a stepping stone to establishing a more advanced musculoskeletal model (Heller et al., 2001a, b, 2003, 2005; Arnold et al., 2009; Trepczynski et al., 2012, 2018; Hutchinson et al., 2015) of the hindlimb in extant and extinct avians to elucidate how birds orchestrate the use of muscles to take to the air by a jump. The current study highlighted the capability for all pelvic muscles to act as an internal/external rotator throughout the RoM of the take-off jump along with the previously described and undoubtedly essential FE (Verstappen et al., 1998). Developing such musculoskeletal models would also offer a means to further explore the hypothesis that IER is indeed a crucial motion that needs to be powered or at least controlled to take to the air, which is supported by the data provided here and previous studies supporting a 3D muscle function of the avian pelvic hindlimb more generally (Hutchinson et al., 2015; Rankin et al., 2016; Allen et al., 2021).

The study conducted here had limitations. Bone scans were based on one specimen of a magpie to inform the skeletal system of the biomechanical model. However, the length of the femur of the scanned specimen (38.8 mm) places its size well within one standard deviation of the mean femoral length (40.2 ± 1.5 mm) measured from a population of 81 magpies in the literature (Tomek and Bochenski, 2000). Furthermore, the mass of the specimen used here (190 g) was very close to the mean mass (188 ± 20g) of the seven magpie specimens analysed by Verstappen et al. (1998) and, thus, the data used here appears to be reasonably representative of a typical magpie. Although the muscle attachment sites were not obtained from the specimen itself, they were informed by the literature (Verstappen et al., 1998) following a careful approach to map the attachments on the surfaces of the 3D CT scanned specimen. Moreover, although the crow, the species informing the locations of the via points, is a different species to the magpie, they are closely related and both belong to the family of Corvidae, within which, hindlimb morphology is very conserved (Verstappen et al., 1998). Furthermore, a detailed sensitivity analysis considering the uncertainty presented by using data from different sources, analysing 972 variations of 14 muscles, ensured that pelvic muscle function was ascertained in a robust manner.

The approach developed here combines state-of the art 3D CT imaging and computer graphics and visualisation techniques with detailed anatomical descriptions of musculoskeletal anatomy of birds obtained in the past (Hudson, 1937) to efficiently establish the 3D representations of the musculoskeletal anatomy. Maximum isometric force of the muscle was calculated from PCSA described in literature (Verstappen et al., 1998), and was scaled by mass to the scanned specimen. This study investigated muscle function throughout of the RoM during the take-off jump which was informed by data from the literature rather than information for the specimen, for which, the 3D skeletal anatomy was derived. However, the data used to inform the RoM was determined using biplanar fluoroscopy (XRoMM) (Provini and Abourachid, 2018), a method currently deemed to constitute the gold standard for determining accurate 3D skeletal kinematic data (Brainerd et al., 2010; Gatesy et al., 2010).

This is the first investigation into the 3D moment-generating capacity of the hindlimb muscles during the take-off jump of a flying bird. Through the sensitivity analysis, eight of the 14 muscles were found to be modelled sufficiently with a single line of action whereas four of the remaining six, owing to their large origin sites, should be modelled with multiple lines of action. Using robust quantitative analysis, our study revealed that while most (11 of 14) muscles acted as either flexor or extensor of the hip, all key pelvic muscles studied here also have the capacity to act as either internal or external rotators. Similarly, 12 muscles possessed at least some ABD capability, thereby revealing the function of the avian hip to be essentially 3D in nature. The ability for all pelvic muscles to act as an internal/external rotator implies that IER might be a crucial motion that needs to be powered or at least controlled to take to the air. Advanced musculoskeletal models of the hindlimb can now use the quantitative evidence on the essential hindlimb muscle function obtained here to reveal in detail how birds orchestrate their muscles to generate the forces necessary to take to the air by a jump.

## Data Availability Statement

The raw data supporting the conclusions of this article will be made available by the authors, without undue reservation.

## Ethics Statement

The animal study was reviewed and approved by University of Southampton Ethics Committee (ERGO ID 21781).

## Author Contributions

EM, CP, NG, and MH: conceptualization and writing–review and editing. EM and MH: methodology, formal analysis, writing–original draft, visualization, and investigation. EM, NG, and MH: data curation. CP, NG, and MH: funding acquisition. All authors contributed to the article and approved the submitted version.

## Conflict of Interest

The authors declare that the research was conducted in the absence of any commercial or financial relationships that could be construed as a potential conflict of interest.
