# Supplementary material for: On the 3D Nature of the Magpie (Aves: Pica pica) Functional Hindlimb Anatomy During the Take-Off Jump
Source: Front Bioeng Biotechnol. 2021 Jun 29;9:676894. doi: 10.3389/fbioe.2021.676894 (PMC8275989; doi:10.3389/fbioe.2021.676894)
Supplement: Supplementary file 1 [file Data_Sheet_1.PDF]

Supplementary Material

1. Description of bone anatomical and joint coordinate systems

Bone local coordinate systems were defined using least squares shape-fitting techniques (Least Squares Geometric Elements library (Forbes, 1989)) applied to select regions of the bone surfaces. Here, derived features such as the femoral head centre, and the axes of the distal femoral, tibiotarsal, tarsometatarsal condyles informed the definition of segment coordinate systems as explained in detail below. Anatomical and joint coordinate systems (ACS and JCS, respectively (Kambic et al., 2014)) were defined for the long bones where ACSs were defined at the proximal end of long bones whilst the JCSs were defined at the distal portion of the bone. Orientation of the coordinate systems followed conventions described in the literature (Kambic et al. (2014), Provini and Abourachid (2018), Fig. S1).

Surfaces defining the spherical regions of the left and right acetabula were isolated (Paraview 5.6.0, Ahrens et al. (2005); Ayachit (2015)). Using Matlab (2018a, The Mathworks, Nantucket, USA) the origin of the pelvic ACS was then defined at the midpoint between the two spheres fitted to isolated regions of the acetabular joint surfaces (lssphere.m v1.0). A midsagittal plane was defined using an iterative approach where the mirrored pelvis surface was registered to its original shape (Chen et al., 2018). The Z axis of the pelvis ACS was the normal of the midsagittal plane pointing from left to right. The Y axis was derived from a Principal Component Analysis (Jolliffe, 2011) of the surface nodes as projection of the 1st principal axis on the midsagittal plane. The pelvic ACS X axis was determined from the cross product of the Y and Z axes. The positive direction of the X axis pointed cranially and the Y axis positive dorsally. The axes of the JCS of the left and right hips were aligned with the axes of the pelvic ACS while their origins were located at the respective femoral centres of rotation.

To define the centre of rotation of the hip, the spherical region of the femoral head was identified (Paraview 5.6.0 Ahrens et al. (2005); Ayachit (2015)) and fitted by a sphere. The X axis direction of the femoral ACS (positive proximally) was defined by determining the centroid line of the femoral shaft to which a straight line was fitted (lls3dline.m v1.0). Its Z axis (positive medially) was defined between the femoral head centre and its projection on to fitted shaft axis. The Y axis of the femoral ACS (positive anteriorly) was determined from the cross product of the X and Z axes. To define the femoral JCS, CT scans of the magpie hindlimb in three different orientations were used to identify the primary axis of rotation using a functional approach (Ehrig et al., 2007; Ehrig and Heller, 2019). The knee centre of rotation was then determined as the midpoint of the medial and lateral intersections of the functional axis of rotation with the bone surface. The functional knee axis of rotation defined the Z axis of the JCS (positive medially). The Y axis of the femoral JCS (positive anteriorly) was determined from the cross-product of the Z axis of the JCS with the X axis of the ACS. The X axis of the femoral JCS (positive proximally) was determined from the cross-product of its Y and Z axes.

The processes for defining the local coordinate systems of the tibiotarsus and tarsometatarsus were the same: the ACS X axis direction (positive proximally) was defined by fitting a line to a centroid axis of the bone shaft. The Z axis of the JCS (positive medially) was defined between the points of intersection of cylinders fitted to the distal medial and lateral condyles with the bone surface (lscylinder.m v1.0). The Y axes (positive anteriorly) of both the ACS and JCS were defined by calculating the cross product between the JCS Z and ACS X axes. JCS X axes (positive proximally)

were defined by calculating the cross product between the JCS Y and Z axes. ACS Z axis (positive medially) was defined by calculating the cross product between the ACS X and Y axes. Tibiotarsal and tarsometatarsal ACS origins were defined at the intersection of the shaft axes with the proximal surface of the respective bone. The origin of the JCS defined as the midpoint of intersections of the Z axis with the bone surface.

1.1 Supplementary Figures

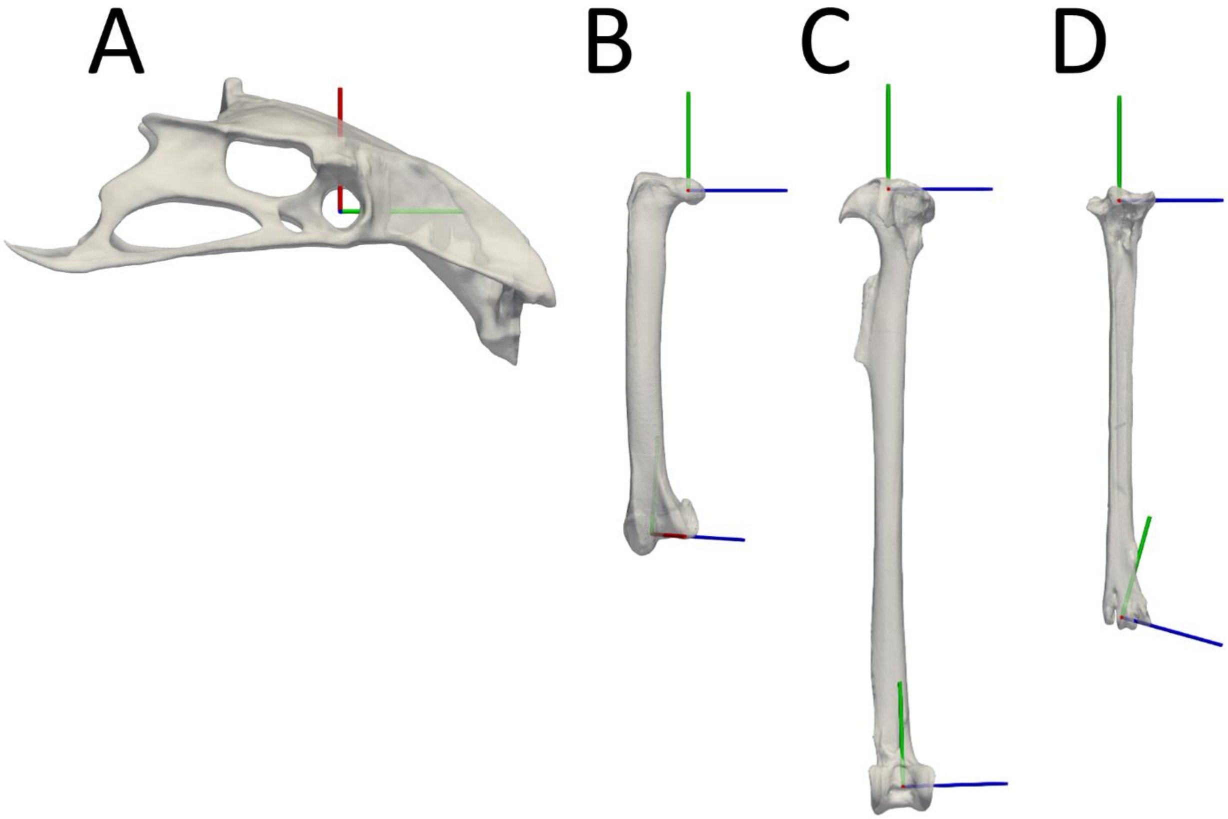

Supplementary Figure 1. Anatomical and joint coordinate systems of the right hindlimb bones of the magpie. For the pelvis (A), the x axis direction (green) points from caudal to cranial, the y axis direction (red) points from ventral to dorsal, and the z axis direction (blue) points from left to right. For the long bones (B to D), the x axis direction points from distal to proximal, the y axis direction points from posterior to anterior, and the z axis direction points from lateral to medial.
